# Sex and age differences in alcohol-attributable mortality in Chile between 2008 and 2022

**DOI:** 10.1016/j.puhip.2026.100798

**Published:** 2026-05-08

**Authors:** José Ruiz-Tagle Maturana, Francisca Román Mella, Álvaro Castillo-Carniglia

**Affiliations:** a Fundación Instituto Profesional DUOC UC, Santiago, Chile; b Millennium Nucleus for the Evaluation and Analysis of Drug Policies (nDP), Chile; c Departamento Nacional de Salud Pública, Facultad de Medicina, Universidad San Sebastián, Santiago, Chile; d Departamento de Psicología, Universidad de la Frontera, Temuco, Chile

**Keywords:** Alcohol, Mortality, Chile

## Abstract

**Objective:**

To analyze trends in alcohol-attributable mortality in Chile from 2008 to 2022 by sex, age, and cause of death.

**Study design:**

Secondary analysis of repeated cross-sectional and administrative data.

**Methods:**

We estimated alcohol-attributable fractions (AAFs) via relative risk functions from published meta-analyses and alcohol consumption data from repeated cross-sectional national drug surveys. Individual deaths were retrieved from official national mortality statistics coded according to the International Classification of Diseases, tenth revision. Uncertainty estimates were generated via Monte Carlo simulations.

**Results:**

In 2008, 14.6% (95% CI: 10.9%, 18.4%) of all deaths were attributable to alcohol, decreasing to 9.6% (95% CI: 7.2%, 12.2%) in 2022. Liver cirrhosis, injuries, and cardiovascular diseases are the leading causes of alcohol-attributable mortality. Males experienced significantly higher mortality rates than women (at least 6 percentage points over the entire period), with injuries being the leading cause among younger people, liver cirrhosis for middle-aged people, and cardiovascular diseases among older people. Among females, liver cirrhosis and cardiovascular diseases were the primary contributors.

**Conclusions:**

Alcohol-attributable mortality in Chile declined between 2008 and 2012 and remained relatively stable thereafter, with persistent disparities across sex and age groups. The results suggest that high-risk drinking patterns continue to play an important role in alcohol-related harm, particularly in younger populations where injuries account for a substantial share of deaths.

## 1 Introduction

Alcohol use is one of the main contributors to the burden of disease globally [1]. It is causally related to more than 200 chronic and acute health conditions, including several types of cancer and cardiovascular, gastrointestinal, and infectious diseases [2]. Alcohol use is also a main contributor to the burden of neuropsychiatric conditions. Alcohol use disorder (AUD) by itself is among the most prevalent psychiatric disorders, with a global estimated prevalence of 8.6% (95% CI: 8.1, 9.1%) for men and 1.7% (95% CI: 1.6, 1.9%) for women in 2016, with a total estimated prevalence of 5.1% (95% CI: 4.9, 5.4%) [3]. Similarly, AUD is highly disabling and is associated with multiple physical and psychiatric comorbidities [4], accounting for approximately 10% of the burden of disease related to substance use and mental disorders [5]. Alcohol consumption, particularly episodes of heavy drinking, is also linked to several external causes of morbidity and mortality, including motor vehicle accidents, falls, drowning, poisonings, intentional self-injuries, and harm to others [[6], [7], [8], [9]].

Chile is the country in the Americas with some of the largest volumes of per capita alcohol consumption [10]. According to the last National Household Surveys on Drug Use of Chile, which includes people aged 12-64 years, the past-month prevalence of alcohol use was 44.3%, with little variation across socioeconomic groups and sexes [11]. The same study revealed that near 50% of people who drank in the past month reported at least one heavy episodic drinking in that same period (5+ drinks on a single occasion for men and 4+ for women), with significant differences across sexes. A study focused on economic costs related to alcohol showed that 0.8% of the gross domestic product (US$2.24 billion in 2017) was related to consequences of alcohol use; of these, 30.1% was due to health-related costs, and 52.2% was due to loss of productivity [12].

While Chile has implemented several policy changes in the last two decades, including an increase in alcohol taxation (3.5 percentage points for spirits and 5.5 percentage points for beer, wine, and ciders), restrictions on marketing, and strengthened drink-driving laws, these measures are not fully aligned with international recommendations regarding the most cost-effective interventions, particularly in terms of their intensity and scope. This may contribute to the persistence of relatively high levels of alcohol consumption in the country [13].

To our knowledge, three studies have estimated alcohol-attributable mortality in Chile [12,14,15]. All of these studies have similar methodologies, which are based on the estimation of the alcohol population attributable fraction to calculate the number of deaths that are totally or partially attributable to alcohol use. The first study was the Chilean Burden of Disease Study, in which the number of deaths attributable to alcohol use was estimated to be 8366 in 2004, corresponding to 9.7% of the total deaths for that year [15]. Consistently, in a subsequent study using 2009 mortality data and a more refined calculation of the population attributable fraction, the estimated number of deaths was 8,750, corresponding to 9.8% (95% CI: 7.0%, 13.0%) of all deaths in that year [14]. In the most recent study, the authors estimated that 13% of the deaths in 2014 were attributable to alcohol use, corresponding to 13,260 deaths that year. Among young (15–29 years of age) men, alcohol use is responsible for up to 50% of all deaths, explained mostly by the large proportion of deaths from external causes, particularly motor vehicle accidents, homicides, and suicides [14].

While previous studies conducted in Chile have provided cross-sectional estimates of alcohol-attributable mortality, they do not capture how these patterns evolve over time. Trend analyses are essential for identifying temporal changes in alcohol-related harm, assessing the potential impact of policy and behavioral shifts, and detecting emerging disparities across population subgroups, as highlighted in comparative risk assessment frameworks. This study seeks to fill this gap by providing a comprehensive analysis of alcohol-attributable mortality trends in Chile between 2008 and 2022, using up-to-date methods for indirect estimation and stratified analyses to identify subpopulations with greater burdens of alcohol consumption.

## 2 Methods

### 2.1 Study design

This is a secondary analysis of repeated cross-sectional and administrative data from 2008 to 2022 in Chile.

### 2.2 Mortality data

Mortality data were sourced from the Department of Health Statistics and Information, which is part of the Chilean Ministry of Health. The dataset includes all deaths registered in the country between 2008 and 2022. Causes of death are classified via the International Classification of Diseases, 10th Revision (ICD-10). The specific ICD-10 codes for each cause are detailed in the supplementary material (Table S1). The mortality datasets include information on sex, age, and primary cause of death.

### 2.3 Alcohol use data

Data on alcohol consumption were obtained from the National Household Surveys on Drug Use of Chile, which has been conducted every two years since 1994. We used surveys from 2008, 2010, 2012, 2014, 2016, 2018, 2020, and 2022. This survey employs a three-stage random sampling design of noninstitutionalized populations at the national and regional levels. Alcohol consumption was estimated using survey weights across each subpopulation of interest (i.e., sex and age groups). To quantify alcohol consumption, we used the first three items of the alcohol use disorder identification tests, which inquire about the frequency and quantity of alcohol use, as well as the frequency of heavy episodic drinking. The daily average number of drinks was then converted to grams of pure alcohol. In the absence of consumption data for individuals older than 64 years, we assumed that the consumption distribution for older age groups was similar to those observed in the oldest age group available.

The survey estimates were then calibrated against the Sustainable Development Goal indicator of total per capita alcohol consumption (15+) provided by the World Health Organization (WHO) [18]. WHO estimates are based on national reports of production, imports, and exports, as well as an estimate of unrecorded alcohol consumption. These values were used as a benchmark for total alcohol consumption, and the proportion not captured by the survey was incorporated as a correction factor. This adjustment accounts for underreporting in self-reported alcohol consumption and rescales the estimated daily grams of pure alcohol for each sex–age group accordingly.

Since the observed distribution of alcohol use can be unstable and present zero observations at specific consumption levels, the empirical distribution was smoothed by fitting a gamma distribution for each sex–age group, from which shape and scale parameters were obtained using *fitdist* function in R [19]. Following the recommendation of several international researchers, we capped the maximum level of alcohol consumption at 150 g per day [[20], [21], [22]].

### 2.4 Statistical analysis

#### 2.4.1 Alcohol-attributable fraction

Alcohol-attributable fractions (AAFs) were estimated using relative risk (RR) functions derived from published meta-analyses of alcohol-related deaths18. These RR functions describe the relationship between alcohol consumption, measured in grams per day, and death risk for each cause related to alcohol. The RR functions were specific to each disease, sex, and age group, reflecting the varying risks posed by different levels of alcohol consumption.

(1) $$\frac{P_{form}\left({RR}_{form}-1\right)\int_{>0}^{150}P_{CD}\left({RR}_{CD}-1\right)}{P_{form}\left({RR}_{form}-1\right)\int_{>0}^{150}P_{CD}\left({RR}_{CD}-1\right)+1}$$

The integral in Equation (1) is evaluated over the distribution of daily alcohol consumption (grams per day). The integral combines the estimated probability density function with the corresponding relative risk function and was approximated numerically using the trapezoidal rule over the range of 0 to 150 g per day. The former drinkers (*form*) were those who had consumed alcohol in the previous year but not within the previous month, and the current drinkers (*CD*) were individuals whose average daily alcohol consumption was between >0 and 150 g.

For ischemic stroke, ischemic heart disease, and injuries, current drinkers were further stratified according to heavy episodic drinking status (HED), because the corresponding relative risk functions distinguish between both patterns. The distinction between HED and non-HED drinkers was incorporated into the modeling framework to align with the structure of the relative risk functions used in previous comparative risk assessment studie [16,17], which differentiate risks by drinking patterns and consumption levels. The 60 g threshold was therefore used only within the integration step, rather than as a direct classification criterion in the survey data (equation (2)).

(2) $$P_{CD}\left(RR-1\right)=\int_{>0}^{60}P_{NHED}\left(x\right){RR}_{NHED}\left(x\right)dx+\int_{>0}^{60}P_{HED}\left(x\right){RR}_{HED}\left(x\right)dx+\int_{60}^{150}P_{HED}\left(x\right){RR}_{HED}\left(x\right)dx$$

#### 2.4.2 Uncertainty estimation

The uncertainty in the AAF estimates was quantified using Monte Carlo simulations with 10,000 iterations [23]. The proportion of former drinkers was simulated via a binomial distribution, whereas the proportion of current drinkers was simulated using a gamma distribution based on empirical data. For the RR functions, we simulated log (RR) values using a normal distribution when a single beta coefficient was used or a multivariate normal distribution when the RR function had multiple beta coefficients. The 2.5 and 97.5 percentiles of the 10,000 simulations were used to generate 95% confidence intervals for the AAFs, accounting for the variability in alcohol consumption and relative risk estimates.

## 3 Results

**Fig. 1:**
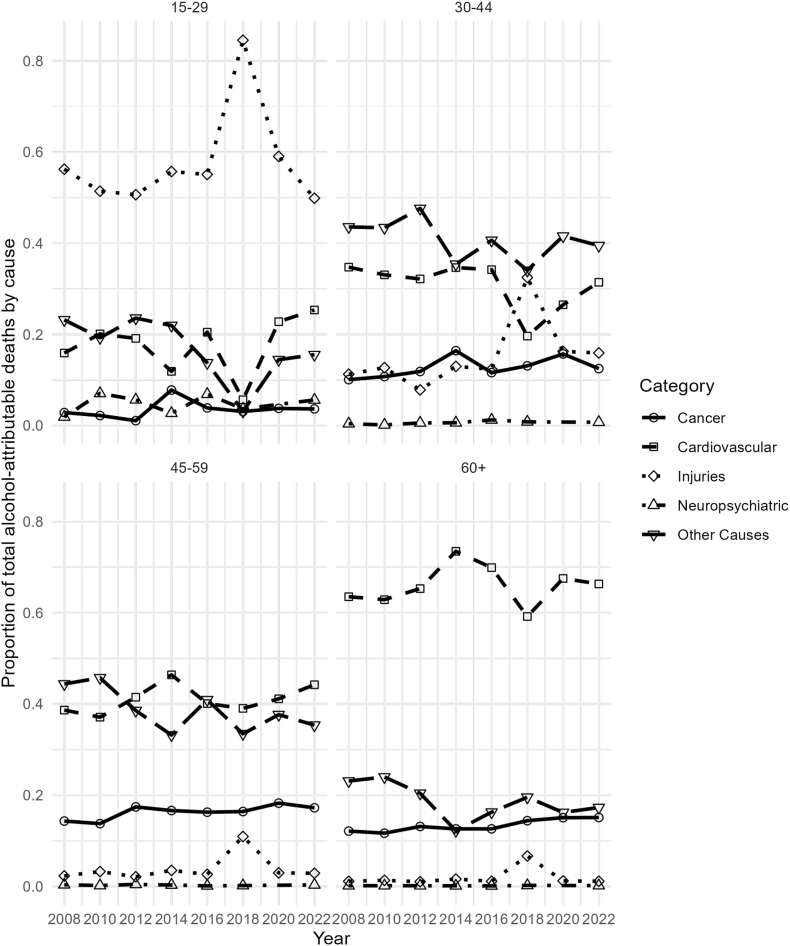
Proportion of alcohol-attributable deaths relative to total deaths by sex and overall, Chile 2008—2022.

Alcohol-attributable mortality accounted for 14.6% (95% CI: 10.9%, 18.4%) of all deaths in 2008, decreasing to 9.6% (95% CI: 7.2%, 12.2%) in 2022 (Fig. 1). Across the study period, men consistently exhibited higher alcohol-attributable death proportions than women. Among men, the proportion declined between 2008 and 2012 and then stabilized at approximately 15%. Among women, the proportion remained lower overall, declining modestly from approximately 10% in 2008 to just below 8% by 2022.

**Fig. 2:**
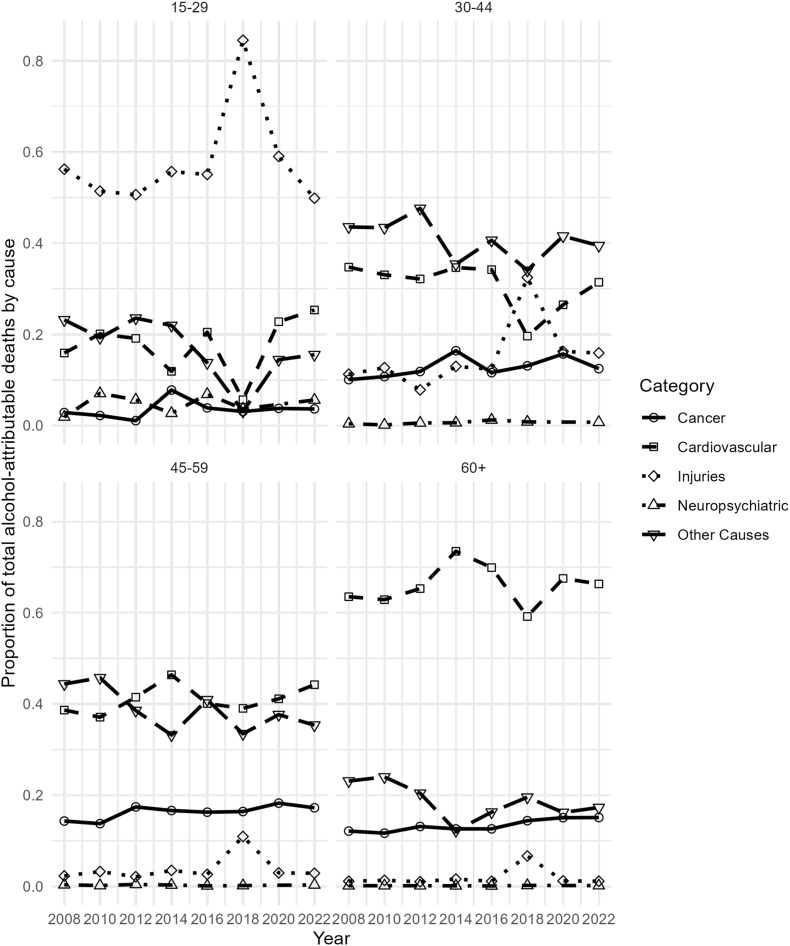
Age-standardized alcohol-attributable mortality rates, Chile 2008 to 2022.

Age-standardized alcohol-attributable mortality rates declined between 2008 and 2012, reaching their lowest point in 2012 (62 deaths per 100,000 population), and then stabilized with minor fluctuations through 2022 (65.4 deaths per 100,000 population) (Fig. 2). Males consistently presented higher mortality rates than females. Among males, rates declined sharply until 2012 and then remained relatively stable, with slight decreases toward the end of the period. In contrast, females experienced a more gradual decline over time, reaching 38.4 deaths per 100,000 population in 2022.

**Fig. 3:**
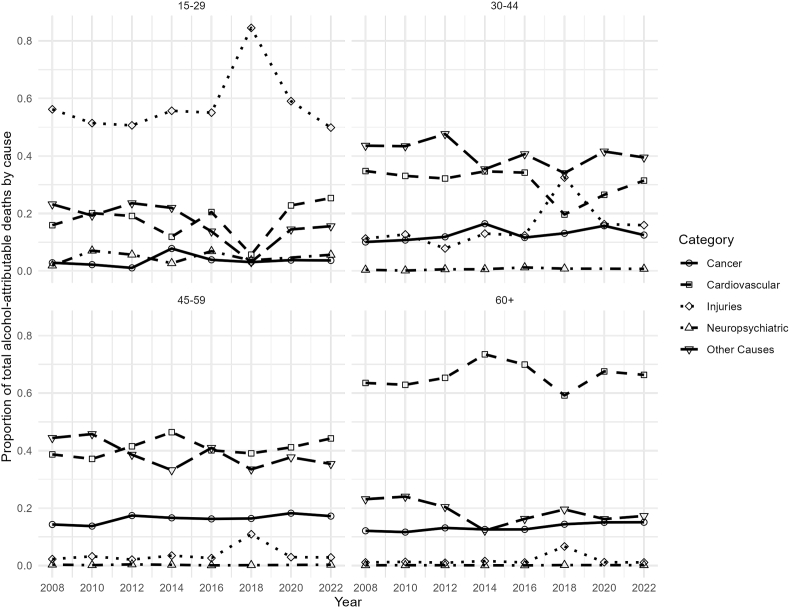
Alcohol-attributable mortality rates by sex and age group, Chile 2008–2022.

Alcohol-attributable mortality rates were consistently higher among men than women across all age groups throughout the study period (Fig. 3). The highest rates were observed in individuals aged 60 years and older, followed by those aged 45–59 and 30–44 years, with the lowest rates in the 15–29 age group. Over time, substantial declines were observed among older age groups, particularly among men aged 60+ and 45–59, whereas younger age groups showed more stable patterns with only slight reductions.

**Fig. 4:**
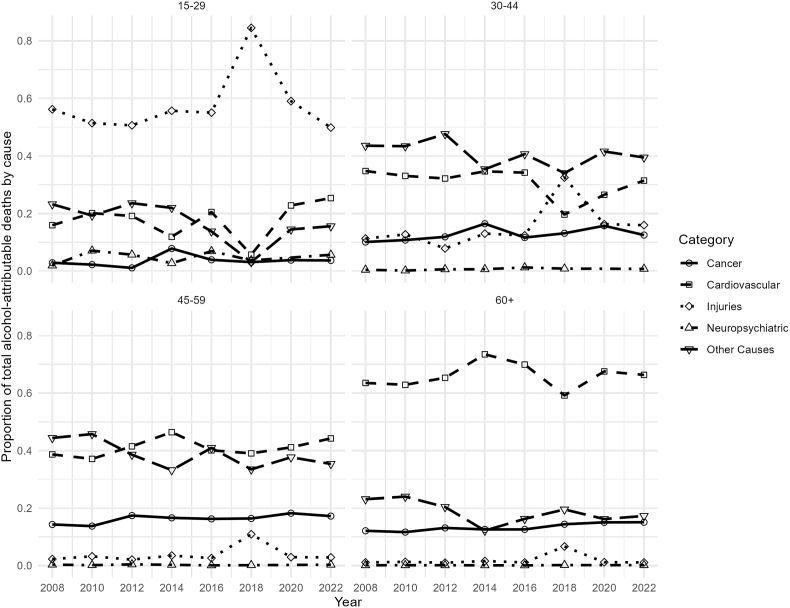
Trends in alcohol-attributable mortality by cause for women, Chile 2008–2022.

Among younger women (15–29 years), injuries accounted for the majority of alcohol-attributable deaths, representing more than 80% of the total (Fig. 4). Motor vehicle accidents increased in 2018, with 120 deaths, but declined to 26 in 2022. Other unintentional injuries also peaked in 2018, then declined in 2022. In middle-aged groups (30–44 and 45–59 years), the proportion of deaths attributed to injuries declined, with “Other Causes" emerging as the predominant category. This category includes infectious and gastrointestinal causes, type 2 diabetes, and liver cirrhosis, the latter being the leading contributor to alcohol-related deaths in these age groups, peaking at 768 in 2008 and decreasing to 516 in 2022, with some fluctuations over time. For older women (60+ years), cardiovascular diseases and “other causes" accounted for the largest proportions of alcohol-attributable deaths (see Supplementary Table S2 for complete counts).

**Fig. 5:**
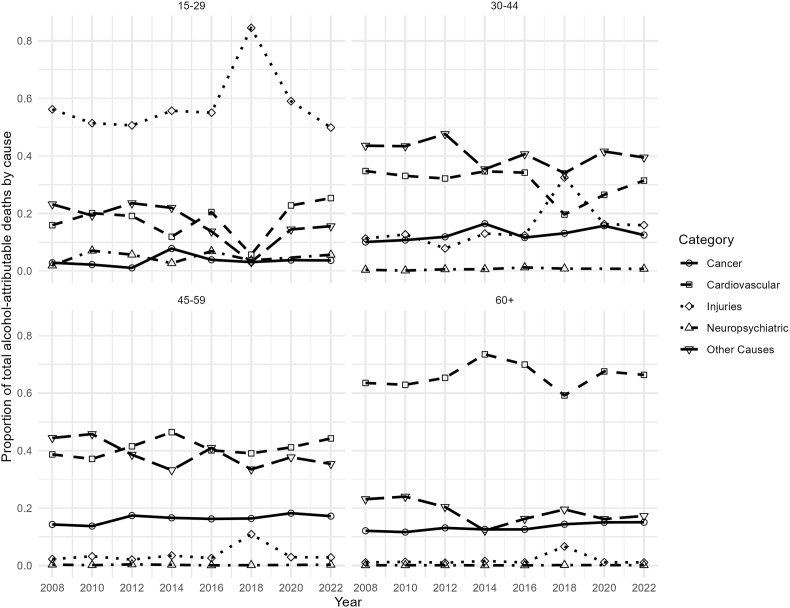
Trends in alcohol-attributable mortality by cause for men, Chile 2008–2022.

Among younger men (15–29 years), injuries were the leading cause of alcohol-attributable mortality throughout the study period (Fig. 5). In middle-aged groups (30–44 and 45–59 years), the contribution of injuries declined over time, with “other causes” becoming increasingly important. Among older men (60+ years), cardiovascular diseases represented the largest contributors to alcohol-attributable mortality (see Supplementary Table S3 for complete counts).

## 4 Discussion

This study analyzes alcohol-attributable mortality trends in Chile from 2008 to 2022. In 2008, 14.6% (95% CI: 10.9%, 18.4%) of all deaths were attributable to alcohol, whereas in 2022, this figure decreased to 9.6% (95% CI: 7.2%, 12.2%). The mortality rate also decreased, reaching its lowest point in 2012 (62 deaths per 100,000 population). From 2012 onward, the trend stabilized with minor fluctuations, peaking slightly in 2018 before declining again through 2022 (65.4 deaths per 100,000 population).

We detected variations across specific causes of alcohol-attributable mortality and demographic subgroups. The burden of alcohol-attributable mortality was consistently greater among males across all age groups, consistent with findings from studies conducted in high-income countries [1,17,24]. In 2022, 12.4% of all male deaths were attributable to alcohol, whereas 6.4% of all female deaths were attributable to alcohol. The mortality rates followed the same pattern. In men, it decreased until 2012 and then stabilized, with minor declines through 2022 (84.4 deaths per 100,000 population), whereas in women, it stabilized in 2012 and reached 38.4 deaths per 100,000 population in 2022. In addition, we found different leading causes of alcohol-related mortality between the sexes. In older men, the leading causes were cardiovascular diseases and liver cirrhosis, whereas in women, cardiovascular diseases led by ischemic heart disease displayed an increasing trend over time. In both sexes, injuries, including motor vehicles and other unintentional injuries, are the main cause of alcohol-attributable death in younger people. We found an elevated alcohol-attributable injury rate in Chilean women in 2018. This can be hypothetically linked to the substantial increase in reported binge drinking during this year. Binge drinking is a well-established risk factor for various types of injuries, including motor vehicle accidents, falls, and other unintentional incidents [25]. However, given the descriptive nature of the data, this temporal overlap should be interpreted with caution, and no direct causal relationship can be inferred. Similar increases in binge drinking among women have been reported in other settings, including the United States [[26], [27]].

After the initial decline in alcohol-attributable mortality between 2008 and 2012, there was an overall stable trend until 2018, followed by a decline in the COVID-19 period. Contrary to findings from other settings, we did not observe an increase in alcohol-attributable mortality during the COVID-19 period [[28], [29], [30], [31], [32], [33], [34], [35], [36], [37]]. Although Chile's mortality coding system prioritizes a single underlying cause of death, potentially leading to some underestimation of alcohol-attributable mortality during the pandemic, this mechanism does not fully explain the observed patterns. Additional analyses separating injury subtypes and non-injury causes (Supplementary Fig. S1) revealed heterogeneous trends. Road injuries declined markedly during 2020–2022, consistent with reduced mobility during pandemic-related restrictions [38], while intentional injuries remained relatively stable. In contrast, declines in alcohol-attributable mortality from chronic conditions predated the COVID-19 period and followed long-term downward trends without clear deviations during 2020–2022.

In discussing the limitations of this study on alcohol-attributable mortality in Chile, several factors should be considered. First, alcohol consumption data were limited to individuals aged 15–64 years, as defined by the national surveys. In the absence of data for older adults, we assumed that the alcohol-attributable fractions for individuals aged 65 years and older were similar to those observed in the oldest age group available in the survey. This may limit generalizability and potentially underestimates the total burden of alcohol-related harm, considering that older adults have specific drinking and mortality patterns [39], particularly due to medication use [40]. Second, although we corrected per capita alcohol consumption with international standards, this correction does not consider differences in subpopulations, including differences across subpopulations (e.g., sex, age, and geographic areas). Third, because Chilean mortality data include only a single underlying cause of death, we relied on indirect estimation methods (i.e., alcohol-attributable fractions), which may introduce uncertainty in the attribution of deaths to alcohol-related causes. Finally, the use of relative risk functions derived from non-local populations represents an additional limitation. These functions are based on large-scale, high-quality evidence and are widely used in Global Burden of Disease and comparative risk assessment studies, ensuring methodological consistency and comparability across settings. However, they may not fully capture region-specific differences in drinking patterns, comorbidities, and healthcare access, which could affect the accuracy of the estimates in the Chilean context.

Future research should prioritize the factors driving alcohol-attributable mortality trends, with a particular focus on the intersection of policy changes, socioeconomic dynamics, and consumption patterns. We also recommend that future studies focus on regional disparities in alcohol-attributable mortality in South America, which is often an overlooked region. Longitudinal studies could explore the impact of specific interventions, such as alcohol taxation, advertising restrictions, and accessibility limitations, on mortality outcomes across demographic subgroups. Additionally, research should aim to develop region-specific relative risk functions tailored to Latin America, as current models rely on global data that may not accurately reflect local epidemiological and consumption profiles. Finally, future studies should address the role of comorbid conditions and polysubstance use in shaping the burden of alcohol-attributable mortality, particularly among older adults.

### 4.1 Conclusion

Alcohol-attributable mortality declined during the early years of the study period and remained relatively stable thereafter. The burden of alcohol-related mortality continues to be concentrated in specific causes and populations, particularly injuries among younger individuals and chronic conditions such as liver cirrhosis and cardiovascular diseases among older adults. Our findings suggest that Chile should target high-risk drinking behaviors, particularly heavy episodic drinking, which has been identified as a key driver of alcohol-related harm and socioeconomic inequality [41].

Strengthening alcohol control policies, particularly those addressing affordability, availability, and harmful drinking patterns [42], may be critical to achieving sustained reductions in alcohol-attributable mortality. In addition, targeted interventions aimed at reducing high-risk behaviors, such as brief interventions targeting drinking and driving, may offer complementary strategies to reduce alcohol-related harm in specific populations [43].

## Ethics statement

Given strict anonymity of collected data, the study did not need authorization of an ethics committee.

## Data statement

The datasets and code used during the current study are available in the Projects’ github repository, for more information, please visit https://doi.org/10.5281/zenodo.18375712.

## Authors' contributions

JRTM conducted the data analysis, interpreted the results, and drafted the manuscript. FR provided substantial revisions to the manuscript. ACC contributed to the study's conception and design and provided significant revisions to the manuscript. All authors reviewed and approved the final version of the manuscript.

## Declaration of Generative AI and AI-assisted technologies in the writing process

During the preparation of this work the authors used GPT-5 to improve writing clarity and coherence. After using this tool, the author(s) reviewed and edited the content as needed and took full responsibility for the content of the publication.

## Funding

This work was supported by 10.13039/501100002850FONDECYT Regular N° 1240138, and by ANID—Millennium Science Initiative Program, no. NCS2021_003.

## Declaration of competing interest

The author is an Editorial Board Member/Editor-in-Chief/Associate Editor/Guest Editor for this journal and was not involved in the editorial review or the decision to publish this article.

The authors declare the following financial interests/personal relationships which may be considered as potential competing interests: None.
