# Supplementary material for: Sex and age differences in alcohol-attributable mortality in Chile between 2008 and 2022
Source: Public Health Pract (Oxf). 2026 May 8;11:100798. doi: 10.1016/j.puhip.2026.100798 (PMC13195772; doi:10.1016/j.puhip.2026.100798)
Supplement: Multimedia component 1 [file mmc1.docx]

Supplemental material

**Table S1. Alcohol-related causes, source of relative risk, and ICD-10 codes**

| **Causes** | **Source** | **ICD-10 Codes** |
| --- | --- | --- |
| **Neuro-psychiatric** |  |  |
| Mental and behavioral disorders due to alcohol | AAF=1 | F10 |
| Degeneration of nervous system due to alcohol | AAF=1 | G312 |
| Epilepsy | Samokhvalov (2010)^1^ | G40-G41 |
| Alcohol polyneuropathy | AAF=1 | G621 |
| **Cardiovascular** |  |  |
| Hypertensive disease | WHO (2018)^2^ | I10-I15 |
| Ischemic heart disease | Rehm (2016)^3^ | I20-I25 |
| Alcoholic cardiomyopathy | AAF=1 | I426 |
| Cardiac arrhythmias | AAF=1 | I48X |
| Hemorrhagic and other non-ischemic stroke | Larsson (2016)^4^ | I60-I62 |
| Ischemic stroke | Rehm (2016)^3^ | I63-I66 |
| **Cancer** |  |  |
| Mouth, oropharynx and laryngeal cancer | Bagnardi (2013)^5^ | C00-C14 |
| Esophageal cancer | Bagnardi (2013)^5^ | C15 |
| Colon cancer | Bagnardi (2013)^5^ | C18 |
| Colorectal cancer | Bagnardi (2013)^5^ | C19X |
| Rectal cancer | Bagnardi (2013)^5^ | C20 |
| Liver cancer | Turati (2014)^6^ | C22 |
| Laryngeal cancer | Bagnardi (2013)^5^ | C32 |
| Breast cancer | Bagnardi (2013)^5^ | C50 |
| **Other causes attributable to alcohol** |  |  |
| Non-insulin-dependent diabetes mellitus | Knott (2015)^7^ | E11 |
| Tuberculosis | Imtiaz (2017)^8^ | A15-A19 |
| HIV/AIDS | Rehm (2017)^9^ | B20-B24 |
| Pneumonia | Samokhvalov (2010)^1^ | J12-J18 |
| Alcoholic gastritis | AAF=1 | K292 |
| Cirrhosis of the liver | WHO (2018)^2^ | K70, K74 |
| Alcohol-induced chronic pancreatitis | AAF=1 | K860 |
| Acute and chronic pancreatitis | Samokhvalov (2015)^10^ | K85, K861 |
| **Injuries** |  |  |
| Intentional self-poisoning by exposure to alcohol | AAF = 1 | X65 |
| Accidental poisoning by exposure to alcohol | AAF = 1 | X45 |
| Poisoning by exposure to alcohol, undermined intent | AAF = 1 | Y15 |
| Motor vehicle accidents | WHO (2018)^2^ | a |
| Accidental poisoning | WHO (2018)^2^ | X40, X46-X49 |
| Falls | WHO (2018)^2^ | W00-W19 |
| Fires | WHO (2018)^2^ | X00-X09 |
| Drowning | WHO (2018)^2^ | W65-W74 |
| Other Unintentional injuries | WHO (2018)^2^ | b |
| Self-inflicted injuries | WHO (2018)^2^ | X60-X64, X66-X84, Y87.0 |
| Homicide | WHO (2018)^2^ | X85-Y09, Y87.1 |
| Other intentional injuries | WHO (2018)^2^ | Y35 |

AAF = Alcohol-Attributable Fraction. WHO = World Health Organization

**a** V021–V029, V031–V039, V041–V049, V092, V093, V123–V129, V133–V139, V143–V149, V194–V196, V203–V209, V213–V219, V223–V229, V233–V239, V243–V249, V253–V259, V263–V269, V273– V279, V283–V289, V294–V299, V304–V309, V314–V319, V324–V329, V334–V339, V344–V349, V354–V359, V364–V369, V374–V379, V384–V389, V394–V399, V404–V409, V414–V419, V424–V429, V434–V439, V444–V449, V454–V459, V464– V469, V474–V479, V484–V489, V494–V499, V504–V509, V514–V519, V524–V529, V534–V539, V544–V549, V554–V559, V564–V569, V574–V579, V584–V589, V594–V599, V604–V609, V614–V619, V624–V629, V634–V639, V644–V649, V654– V659,

V664–V669, V674–V679, V684–V689, V694–V699, V704–V709, V714–V719, V724–V729, V734–V739, V744–V749, V754–V759, V764–V769, V774–V779, V784–V789, V794–V799, V803–V805, V811, V821, V830–V833, V840–V843, V850– V853, V860–V863, V870–V878, V892. **b** W20-W64, W 75-W99, X10-X39, X50-X59, Y40-Y86, Y88, Y89 and V series except for the codes listed in note a.

**Table S2. Alcohol-Attributable Mortality for female 15-to 65 years old between 2008 to 2018**

| **Causes** | **2008** | | **2010** | | **2012** | | **2014** | | **2016** | | **2018** | |
| --- | --- | --- | --- | --- | --- | --- | --- | --- | --- | --- | --- | --- |
|  | **n** | **95%CI** | **n** | **95%CI** | **n** | **95%CI** | **n** | **95%CI** | **n** | **95%CI** | **n** | **95%CI** |
| **Neuro-Psychiatric** |  |  |  |  |  |  |  |  |  |  |  |  |
| **Mental and Behavioral disorders** | 13 | AAF = 1 | 10 | AAF = 1 | 11 | AAF = 1 | 5 | AAF = 1 | 4 | AAF = 1 | 4 | AAF = 1 |
| **Degeneration of nervous system** | 0 | AAF = 1 | 3 | AAF = 1 | 1 | AAF = 1 | 1 | AAF = 1 | 0 | AAF = 1 | 1 | AAF = 1 |
| **Epilepsy** | 3 | 2;4 | 4 | 3;5.5 | 4 | 3;5 | 6 | 4; 8 | 3 | 2;4 | 5 | 4;7 |
| **Cardiovascular** |  |  |  |  |  |  |  |  |  |  |  |  |
| **Hypertensive Disease** | 10 | 2;21 | 9 | 1;18 | 8 | 1;17 | 18 | 3;37 | 10 | 2;21 | 11 | 2;23 |
| **Ischemic Heart Disease** | 358 | 291;420 | 344 | 280;402 | 356 | 289;417 | 389 | 316;455 | 373 | 303;437 | 387 | 314;453 |
| **Cardiomyopathy** | 1 | AAF = 1 | 0 | AAF = 1 | 0 | AAF = 1 | 0 | AAF = 1 | 1 | AAF = 1 | 0 | AAF = 1 |
| **Hemorrhagic stroke** | 353 | 238;499 | 316 | 208;461 | 298 | 198;429 | 305 | 187;456 | 289 | 199;403 | 292 | 196;411 |
| **Ischemic stroke** | 20 | 14;27 | 15 | 11;21 | 13 | 9;18 | 13 | 9;17 | 10 | 7;14 | 10 | 7;13 |
| **Cancer** |  |  |  |  |  |  |  |  |  |  |  |  |
| **Mouth, oropharynx** | 4 | 3;5 | 3 | 2;4 | 3 | 2;4 | 3 | 2;4 | 5 | 4;6 | 5 | 4;6 |
| **Esophageal** | 5 | 4;6 | 4 | 3;5 | 2 | 2;3 | 5 | 4;6 | 4 | 3;5 | 5 | 4;6 |
| **Colon, Rectal and Colorectal** | 13 | 10;16 | 12 | 10;15 | 13 | 10;15 | 19 | 14;23 | 15 | 12;18 | 23 | 18;28 |
| **Liver** | 56 | 53;58 | 45 | 43;48 | 50 | 47;53 | 53 | 50;56 | 63 | 60;66 | 63 | 61;66 |
| **Laryngeal** | .5 | .4;.6 | .8 | .7;1 | 0.6 | .5;.7 | 1 | .8;1.2 | 0.6 | .5;.7 | 0.6 | .5;.7 |
| **Breast** | 35 | 27;43 | 34 | 26;42 | 29 | 22;37 | 46 | 37;57 | 33 | 27;41 | 45 | 35;55 |
| **Other causes** |  |  |  |  |  |  |  |  |  |  |  |  |
| **Diabetes Mellitus** | -3 | -8;1 | -4 | -9;1 | -5 | -12;2 | -12 | -21;-3 | -5 | -13;3 | -3 | -7;1 |
| **Tuberculosis** | 2 | .3;4 | 4 | .6;8 | 2 | .4;5 | 3 | .6;6 | 2 | .4;5 | 3 | .5;6 |
| **HIV/AIDS** | .4 | .2;.7 | .5 | .3;.8 | .4 | .2;.7 | .9 | .5;1.4 | .4 | .2;.7 | .6 | .3;.9 |
| **Pneumonia** | 3 | .5;5 | 2 | .4;4 | 2 | .5;4 | 4 | .7;7 | 3 | .5;5 | 4 | .8;8 |
| **Alcoholic gastritis** | 0 | AAF = 1 | 0 | AAF = 1 | 0 | AAF = 1 | 0 | AAF = 1 | 1 | AAF = 1 | 0 | AAF = 1 |
| **Liver Cirrhosis** | 297 | 283;312 | 273 | 258;289 | 256 | 242;272 | 267 | 251;283 | 302 | 289;316 | 262 | 249;276 |
| **Alcohol-induced chronic pancreatitis** | 0 | AAF = 1 | 0 | AAF = 1 | 0 | AAF = 1 | 0 | AAF = 1 | 0 | AAF = 1 | 0 | AAF = 1 |
| **Acute pancreatitis** | 19 | 17;20 | 19 | 18;21 | 12 | 11;13 | 13 | 11;15 | 16 | 15;18 | 15 | 14;16 |
| **Injuries** |  |  |  |  |  |  |  |  |  |  |  |  |
| **Intentional self poisoning** | 0 | AAF = 1 | 0 | AAF = 1 | 0 | AAF = 1 | 0 | AAF = 1 | 0 | AAF = 1 | 0 | AAF = 1 |
| **Accidental poisoning** | 3 | 2;4 | 3 | 2;4 | 1 | 1;2 | 3 | 2;4 | 2 | 1;3 | 5 | 4;6 |
| **Motor vehicle accident** | 30 | 16;45 | 21 | 11;33 | 19 | 10;30 | 26 | 14;40 | 29 | 16;46 | 67 | 40;93 |
| **Falls** | 3 | 2;4 | 4 | 3;5 | 4 | 3;5 | 7 | 6;8 | 5 | 4;6 | 11 | 9;12 |
| **Fires** | 11 | 9;13 | 7 | 6;9 | 5 | 4;6 | 13 | 10;15 | 6 | 5;7 | 12 | 10;14 |
| **Drowning** | 8 | 7;10 | 5 | 4;6 | 2 | 1;3 | 6 | 5;7 | 3 | 2;4 | 9 | 8;10 |
| **Other unintentional injuries** | 20 | 17;24 | 42 | 34;51 | 10 | 8;12 | 35 | 29;41 | 21 | 17;25 | 57 | 49;64 |
| **Homicide** | 18 | 15;21 | 19 | 15;23 | 14 | 11;17 | 23 | 19;27 | 15 | 13;18 | 20 | 17;22 |

Table S2 shows that liver cirrhosis consistently contributed the greatest number of alcohol-attributable deaths among women, peaking at 768 in 2008 and decreasing to 516 in 2022, with some fluctuations over time. Injuries, particularly motor vehicles and other unintentional injuries were significant contributors among younger women. Motor vehicle accidents increased in 2018, with 120 deaths, but declined to 26 in 2022. Other unintentional injuries also peaked in 2018 before declining in 2022. Among cardiovascular causes, ischemic heart disease was the leading contributor, showing an increasing trend. Compared with other categories, neuropsychiatric causes contribute fewer deaths, with mental and behavioral disorders due to alcohol showing a slight increase toward 2022.

**Table S3. Alcohol-Attributable Mortality for males 15-to 65 years old between 2008 to 2018**

| **Causes** | **2008** | | **2010** | | **2012** | | **2014** | | **2016** | | **2018** | |
| --- | --- | --- | --- | --- | --- | --- | --- | --- | --- | --- | --- | --- |
|  | **n** | **95%CI** | **n** | **95%CI** | **n** | **95%CI** | **n** | **95%CI** | **n** | **95%CI** | **n** | **95%CI** |
| **Neuro-Psychiatric** |  |  |  |  |  |  |  |  |  |  |  |  |
| **Mental and Behavioral disorders** | 161 | AAF = 1 | 118 | AAF = 1 | 115 | AAF = 1 | 67 | AAF = 1 | 72 | AAF = 1 | 77 | AAF = 1 |
| **Degeneration of nervous system** | 14 | AAF = 1 | 23 | AAF = 1 | 13 | AAF = 1 | 7 | AAF = 1 | 6 | AAF = 1 | 6 | AAF = 1 |
| **Epilepsy** | 9 | 7;12 | 8 | 6;11 | 7 | 5;9 | 11 | 8; 15 | 7 | 5;9 | 11 | 8;14 |
| **Cardiovascular** |  |  |  |  |  |  |  |  |  |  |  |  |
| **Hypertensive Disease** | 60 | 38;84 | 48 | 30;70 | 50 | 31;72 | 75 | 48;105 | 57 | 36;81 | 52 | 33;74 |
| **Ischemic Heart Disease** | 1192 | 968;1397 | 1158 | 940;1356 | 1175 | 954;1376 | 1324 | 1075;1551 | 1209 | 982;1416 | 1240 | 1007;1453 |
| **Cardiomyopathy** | 9 | AAF = 1 | 4 | AAF = 1 | 7 | AAF = 1 | 5 | AAF = 1 | 8 | AAF = 1 | 7 | AAF = 1 |
| **Hemorrhagic stroke** | 427 | 218;680 | 363 | 189;581 | 333 | 170;541 | 412 | 193;677 | 325 | 179;500 | 304 | 176;457 |
| **Ischemic stroke** | 24 | 16;32 | 24 | 16;32 | 19 | 13;26 | 21 | 15;29 | 23 | 16;31 | 20 | 13;26 |
| **Cancer** |  |  |  |  |  |  |  |  |  |  |  |  |
| **Mouth, oropharynx** | 27 | 24;30 | 25 | 21;28 | 25 | 22;28 | 24 | 21;26 | 26 | 22;29 | 32 | 28;36 |
| **Esophageal** | 36 | 29;43 | 25 | 20;30 | 27 | 22;33 | 25 | 20;29 | 25 | 20;30 | 26 | 21;31 |
| **Colon, Rectal and Colorectal** | 94 | 89;98 | 87 | 82;91 | 86 | 81;91 | 113 | 106;119 | 137 | 131;143 | 157 | 150;163 |
| **Liver** | 62 | 59;65 | 57 | 53;60 | 73 | 68;77 | 59 | 56;63 | 74 | 70;77 | 75 | 71;78 |
| **Laryngeal** | 15 | 12;.18 | 10 | 8;12 | 13 | 10;16 | 12 | 9;14 | 8 | 6;10 | 11 | 9;13 |
| **Other causes** |  |  |  |  |  |  |  |  |  |  |  |  |
| **Diabetes Mellitus** | 15 | 11;24 | 14 | 12;21 | 19 | 14;29 | 17 | 13;25 | 17 | 15;23 | 15 | 14;18 |
| **Tuberculosis** | 48 | 9;85 | 43 | 8;80 | 47 | 8;87 | 36 | 7;66 | 31 | 5;59 | 33 | 6;62 |
| **HIV/AIDS** | 18 | 11;26 | 17 | 10;25 | 17 | 10;25 | 21 | 12;30 | 18 | 11;27 | 10 | 6;14 |
| **Pneumonia** | 28 | 6;51 | 30 | 6;57 | 23 | 5;44 | 30 | 6;57 | 25 | 5;47 | 29 | 6;54 |
| **Alcoholic gastritis** | 2 | AAF = 1 | 3 | AAF = 1 | 0 | AAF = 1 | 0 | AAF = 1 | 3 | AAF = 1 | 3 | AAF = 1 |
| **Liver Cirrhosis** | 1361 | 1262;1459 | 1285 | 1186;1383 | 1195 | 1097;1293 | 1284 | 1185;1385 | 1289 | 1195;1386 | 1063 | 988;1143 |
| **Alcohol-induced chronic pancreatitis** | 1 | AAF = 1 | 0 | AAF = 1 | 0 | AAF = 1 | 0 | AAF = 1 | 0 | AAF = 1 | 0 | AAF = 1 |
| **Acute pancreatitis** | 46 | 35;58 | 39 | 31;50 | 38 | 29;49 | 50 | 37;63 | 39 | 31;49 | 40 | 32;50 |
| **Injuries** |  |  |  |  |  |  |  |  |  |  |  |  |
| **Intentional self poisoning** | 0 | AAF = 1 | 0 | AAF = 1 | 0 | AAF = 1 | 0 | AAF = 1 | 0 | AAF = 1 | 0 | AAF = 1 |
| **Accidental poisoning** | 22 | 20;24 | 11 | 10;12 | 9 | 8;10 | 10 | 9;11 | 14 | 13;15 | 20 | 19;22 |
| **Motor vehicle accident** | 126 | 93;163 | 95 | 69;125 | 89 | 64;117 | 105 | 78;136 | 104 | 77;134 | 100 | 74;128 |
| **Falls** | 98 | 89;105 | 82 | 73;89 | 91 | 81;100 | 81 | 73;88 | 75 | 68;83 | 78 | 72;83 |
| **Fires** | 65 | 59;70 | 93 | 84;102 | 51 | 46;56 | 52 | 47;57 | 45 | 41;50 | 48 | 45;51 |
| **Drowning** | 164 | 150;175 | 112 | 101;123 | 97 | 87;107 | 96 | 87;104 | 98 | 88;106 | 109 | 101;116 |
| **Other unintentional injuries** | 394 | 361;423 | 382 | 343;418 | 281 | 252;309 | 413 | 373;448 | 295 | 265;323 | 544 | 503;578 |
| **Homicide** | 308 | 284;329 | 280 | 253;304 | 243 | 219;265 | 315 | 286;341 | 265 | 240;287 | 200 | 186;213 |

Table S3 displays alcohol-attributable mortality trends among men aged 15-65 years, revealing considerably higher mortality rates than in females across nearly all causes. Liver cirrhosis was consistently the leading contributor to alcohol-related deaths, but with fluctuating numbers, from 3,590 in 2008 to 2,938 in 2022. Injuries were prominent, with road injuries declining notably, whereas unintentional injuries peaked in 2018, with 1,146 alcohol-attributable deaths, and then decreased to 708 by 2022. Intentional injuries also exhibited a steady decline. Neuropsychiatric causes, particularly mental and behavioral disorders due to alcohol, fluctuated, with 263 deaths in 2008 and 256 in 2022. The prevalence of alcohol-attributable cancers, such as colon and rectum cancer and liver cancer, has shown an increasing trend, increasing from 210 in 2008 to 418 in 2022 for colorectal cancers and from 178 to 272 for liver cancers. Among cardiovascular causes, ischemic heart disease consistently accounted for the greatest number of deaths, increasing from 1,192 in 2008 to 1,470 in 2022.

**Figure S1. Trends in alcohol-attributable mortality rates by injury subtype and non-injury causes, Chile 2008–2022**


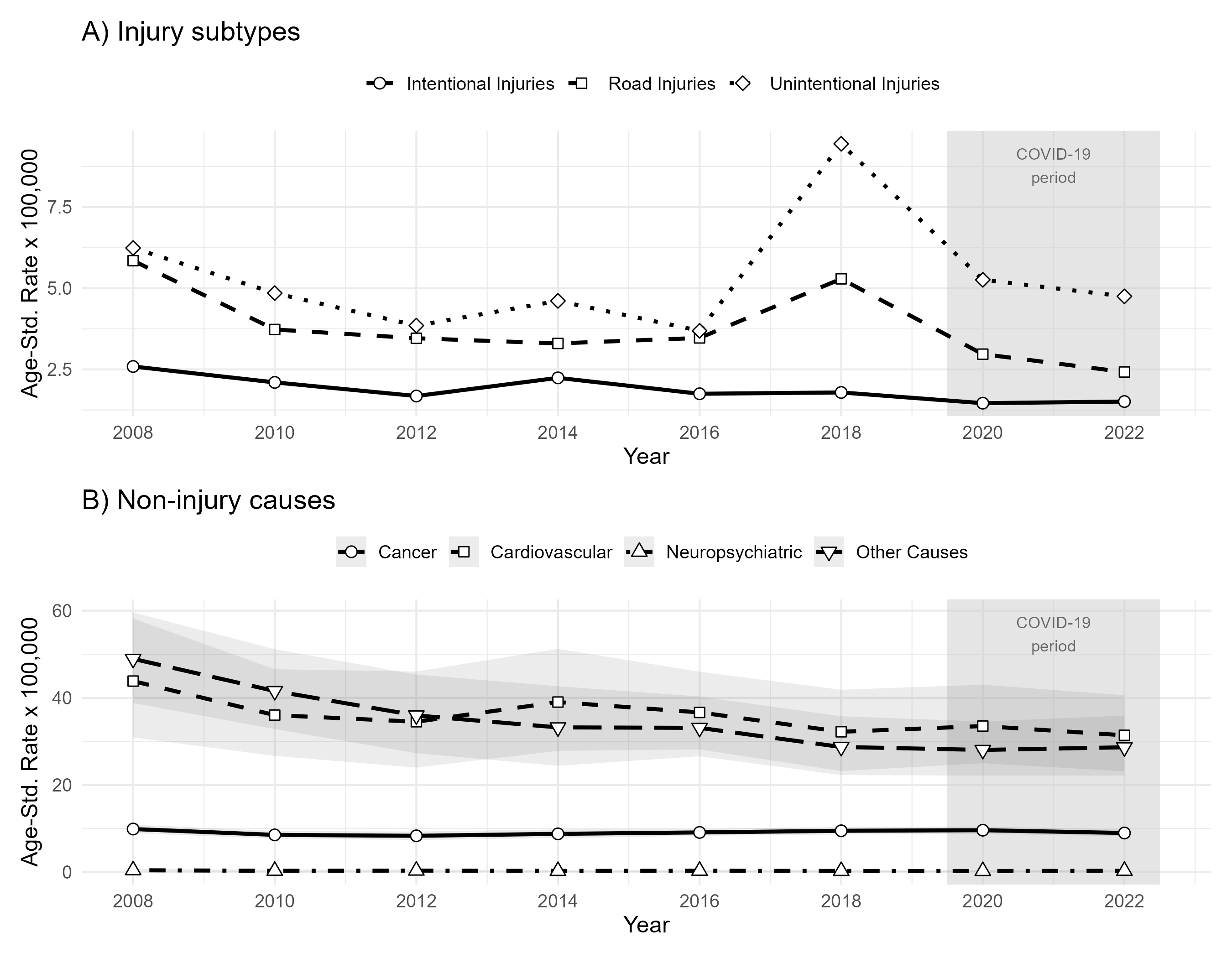


The line plot presented in Figure S1 shows age-standardized alcohol-attributable mortality rates by cause subtype from 2008 to 2022. Panel A shows injury subtypes, including road injuries, unintentional injuries, and intentional injuries. Road injuries declined markedly during the COVID-19 period (2020–2022), consistent with reduced mobility, while intentional injuries remained relatively stable over time. Unintentional injuries exhibited greater variability, including a notable peak in 2018.

Panel B presents non-injury causes, including cancer, cardiovascular diseases, neuropsychiatric conditions, and other causes. These categories show a general downward trend over the study period, with no clear deviation during the COVID-19 years relative to pre-existing trends.

**BIBLIOGRAPHY**

1. Samokhvalov AV, Irving H, Mohapatra S, Rehm J. Alcohol consumption, unprovoked seizures, and epilepsy: A systematic review and meta-analysis. *Epilepsia*. 2010;51(7):1177-1184. doi:10.1111/j.1528-1167.2009.02426.x

2. World Health Organization. *Global Status Report on Alcohol and Health 2018*.; 2018. https://apps.who.int/iris/handle/10665/274603

3. Rehm J, Imtiaz S. A narrative review of alcohol consumption as a risk factor for global burden of disease. *Subst Abuse Treat Prev Policy*. 2016;11(1):37. doi:10.1186/s13011-016-0081-2

4. Larsson SC, Wallin A, Wolk A, Markus HS. Differing association of alcohol consumption with different stroke types: a systematic review and meta-analysis. *BMC Med*. 2016;14(1):178. doi:10.1186/s12916-016-0721-4

5. Bagnardi V, Rota M, Botteri E, et al. Light alcohol drinking and cancer: a meta-analysis. *Ann Oncol*. 2013;24(2):301-308. doi:10.1093/annonc/mds337

6. Turati F, Galeone C, Rota M, et al. Alcohol and liver cancer: a systematic review and meta-analysis of prospective studies. *Ann Oncol*. 2014;25(8):1526-1535. doi:10.1093/annonc/mdu020

7. Knott C, Bell S, Britton A. Alcohol Consumption and the Risk of Type 2 Diabetes: A Systematic Review and Dose-Response Meta-analysis of More Than 1.9 Million Individuals From 38 Observational Studies. *Diabetes Care*. 2015;38(9):1804-1812. doi:10.2337/dc15-0710

8. Imtiaz S, Shield KD, Roerecke M, Samokhvalov AV, Lönnroth K, Rehm J. Alcohol consumption as a risk factor for tuberculosis: meta-analyses and burden of disease. *Eur Respir J*. 2017;50(1). doi:10.1183/13993003.00216-2017

9. Rehm J, Gmel Sr GE, Gmel G, et al. The relationship between different dimensions of alcohol use and the burden of disease—an update. *Addiction*. 2017;112(6):968-1001. doi:10.1111/add.13757

10. Samokhvalov AV, Rehm J, Roerecke M. Alcohol Consumption as a Risk Factor for Acute and Chronic Pancreatitis: A Systematic Review and a Series of Meta-analyses. *eBioMedicine*. 2015;2(12):1996-2002. doi:10.1016/j.ebiom.2015.11.023
